# Nutritional profile and food consumption of the riverside community assisted by the Humanitarian Assistance Program Doutores das Águas

**DOI:** 10.1016/j.pmedr.2025.103283

**Published:** 2025-10-18

**Authors:** Patrícia Colombo-Souza, Julia de Macedo Moura Silva, Bruna da Silva Miranda, Ana Paula Ribeiro, Marco Antonio Zonta

**Affiliations:** a Post Graduate Program in Health Science, Santo Amaro University (Unisa), Rua Prof. Enéas de Siqueira Neto, 340, Zip code: 04829-300, São Paulo, Brazil; b Nutrition Course, Santo Amaro University (Unisa), Rua Prof. Enéas de Siqueira Neto, 340, Zip code: 04829-300, São Paulo, Brazil; c Nutrition Course and Post Graduate Program in Health Science, Santo Amaro University (Unisa), Rua Prof. Enéas de Siqueira Neto, 340, Zip code: 04829-300, São Paulo, Brazil; d Inside Health Group. Associate Researcher at Butantan Institute, São Paulo University (USP), Av. Vital Brasil, 1500, Zipcode: 05503-900, São Paulo, Brazil

**Keywords:** food consumption, Amazon, food insecurity, Riverside community, nutritional status

## Abstract

**Objective:**

To analyze the nutritional profile and its correlation with food consumption patterns among riverside communities in Roraima and Amazonas states.

**Methods:**

A cross-sectional study (April 2019) was performed, including 160 adults from 120 families across 12 communities. Data collection combined 24-h dietary recalls, food frequency questionnaires, and anthropometric measurements. The Kruskal-Wallis analysis of variance was applied using IBM SPSS V.28 software. Nutrient intake was analyzed and compared to Dietary Reference Intakes using AVANUTRI software.

**Results:**

The sample (60.6 % female, mean age 40.9 years) showed a 59.6 % prevalence of overweight/obesity. Diets were high in oils (1.7 L/person/month) and sugar (2.78 kg/person/month), exceeding recommendations by 30–33 %, while fiber intake was critically low (10.9 g/day). Micronutrient deficiencies (vitamins A, C, D, iron, zinc) were widespread. Despite high consumption of ultra-processed foods, caloric and macronutrient intake did not differ significantly by BMI (*p* > 0.05).

**Conclusion:**

Riverside communities face a dual burden of obesity and micronutrient deficiencies, driven by declining traditional diets and increasing ultra-processed food dependence. Culturally adapted interventions are urgently needed to enhance dietary diversity, reduce consumption of processed foods, and bolster local food security.

## Highlights

• High obesity/overweight (59.6 %) with micronutrient deficiencies and food insecurity.
• Caloric excess and critical nutrient shortfalls fracture the local food system.
• Critically low fiber intake (10.9 g/d) amid traditional foods decline.
• High ultra-processed food consumption: 89.2 % sweets/sodas, 58.4 % meats.
• Higher obesity in women (23.2 %) than men (17.5 %) reflects gendered roles.

## 1 Introduction

The concept of food security, which was previously limited to adequate supply, has been expanded to encompass universal access to food, nutritional aspects, and, consequently, the quality and biological value of foods (Martins et al., 2023; Pedraza and de Menezes, 2015). However, data generated on food intake are fundamental for establishing the health conditions of a population, helping to evaluate the associations between diet, nutrition, and health, detect nutrient deficiencies, and characterize the population's risk and vulnerability levels (Steluti et al., 2020; Sorić et al., 2021).

Nutritional habits, which encompass food choices, are influenced by a complex interplay of personal, cultural, socioeconomic, biological, and toxicological factors. These habits work as automatic behaviors that establish a connection between the context in which the person is found and their dietary responses. Although helpful in simplifying decision-making in repetitive situations, these habits are resistant to change (Henchion et al., 2021). In this scenario, the “mix” of factors that influence food choices can be reconfigured to adapt to new emerging priorities. This reassessment may lead to changes in the reasons for food choices and, consequently, in the overall quality of the diet (Wood et al., 2022).

Riverside communities, inhabitants of the vicinity of rivers, are priorities for the National Policy on Food and Nutrition Security of Brazil and are represented by artisanal fishermen and individuals who practice extractivism (who sustainably gather wild forest and river products**)**; they live on stilts, grow crops for their own consumption, and have fishing as their primary means of subsistence (Mercado et al., 2015). Despite the wealth of natural resources, resulting from the enormous diversity of fish and fruits available in the region, riverside communities face high levels of poverty and a low quality of life compared to the national average (Gama et al., 2018).

Socioeconomic development in northern Brazil has significantly altered the dietary practices of riverside populations, challenging traditional food patterns. Access to food purchased in markets, especially in areas closer to urban centers, has led to an increase in the consumption of processed foods and products, such as sugar and beef (Gama et al., 2022). By examining how this dietary transition affects nutritional status, dietary diversity, and health outcomes (e.g., micronutrient deficiencies or diet-related chronic diseases), the current study aims to identify challenges and generate evidence for improving food security and nutritional health in these communities (Silveira et al., 2023a).

The scarcity of research addressing the food realities of riverside populations represents a significant gap in understanding the changes in dietary patterns that occur in response to economic and social development in the region. Thus, the current study aimed to analyze the nutritional status of the population, defined and assessed through anthropometric indicators, as well as the nutritional profile determined by food acquisition and consumption patterns, among riverside communities in Roraima and Amazonas states. Participants are beneficiaries of the *Doutores das Águas Program*—a private non-profit (OSCIP) founded in 2011 by fishing industry entrepreneurs. The program provides mobile medical/dental care and socio-environmental education to isolated Amazonian communities, creating a critical context for assessing how community-led interventions intersect with dietary changes.

## 2 Methods

### 2.1 Study design and setting

A cross-sectional population-based study was conducted in April 2019 among riverside communities in the Amazonian states of Roraima and Amazonas, Brazil. The Amazon region spans 5.2 million km^2^ (61 % of Brazil's territory) (Secretaeria et al., 2023). It includes diverse rural populations, such as self-identified Indigenous groups, caboclos (traditional populations of mixed Indigenous and European ancestry), and quilombolas (descendants of Afro-Brazilian negro communities), many residing in remote riverside areas with a limited infrastructure (Secretaeria et al., 2023). Although data collection was carried out in 2019, its scientific validity remains high due to the absence of subsequent nutritional interventions targeting these specific communities. Data collection in the following years was halted by the COVID-19 pandemic (2020−2021), which made access to these remote areas impossible. A subsequent expedition in 2022, led by the same team, focused on non-dietary health parameters, confirming that the underlying dietary patterns and food security context had not changed.

### 2.2 Sample selection and population

The sample size was calculated to accommodate the study's multiple primary outcomes (food consumption, nutritional status, and food acquisition). As no prior data on key outcomes (e.g., prevalence of ultra-processed food consumption) are available for this specific population, a conservative event prevalence of 50 % was adopted to ensure maximum sample variability. The calculation employed a 5 % margin of error and a 95 % confidence level, resulting in a minimum of 133 participants. After incorporating a 20 % adjustment for potential non-responses and losses, the final sample targeted 160 participants from 120 families across 12 communities. Probabilistic cluster sampling was employed, where households were randomly selected in collaboration with community leaders, alternating between residences based on participant availability. The inclusion criteria were adults aged 18 years or older, who resided in the communities, and who provided informed consent.

### 2.3 Data collection

Data were collected through home interviews using a structured questionnaire that included demographic information, food access strategies (e.g., reliance on local resources, economic constraints, and coping mechanisms), and a dietary assessment.

Food acquisition was defined as the origin of food consumed by families (e.g., own production/extractivism, fishing, purchase from local commerce). Food consumption patterns were assessed using two tools: a 24-h dietary recall and a Food Frequency Questionnaire (FFQ). The 24-h dietary recall captured detailed information on consumption over the previous 24 h, including the methods used for food preparation. Portion sizes were estimated in collaboration with the interviewees using their own household utensils (e.g., cups, spoons, bowls, and glasses) as visual references to quantify the amounts consumed; these estimates were subsequently converted to grams or milliliters.

The FFQ was applied at the household level to the person primarily responsible for food acquisition and/or meal preparation. This respondent reported the habitual consumption frequency of foods for the entire family unit, reflecting the common dietary patterns of the household as a whole. The FFQ categorizes foods into nine groups (cereals/pasta, vegetables, oils, fruits, legumes, meats, dairy, sweets, and processed meats) and records consumption frequency using the following categories: daily (≥1 time/day), weekly (1–6 times/week), monthly (1–3 times/month), or rarely/never (≤1 time/month).

For the evaluation of food consumption, the per capita recommendations of the World Health Organization (WHO) were considered for sugar intake (25-30 g/day) (Guideline: Sugars Intake for Adults and Children, 2015) and oil consumption (16 mL - 25-30 g/day), according to the Brazilian Society of Cardiology's position on fat consumption and cardiovascular health (Izar et al., 2021).

To enhance the dietary data, during the household visits, the researchers also conducted structured direct observations. These observations took place concurrently with the 24-h dietary recall interviews, which lasted approximately 20–40 min per household. Researchers used a standardized checklist to note contextual factors such as the types of food staples visibly available in the home, cooking facilities, and the use of ultra-processed food packaging.

Dietary composition was analyzed using AVANUTRI software, with nutrient intake compared to Dietary Reference Intakes (DRIs) (Onis, 2015), considering the nutrient and energy needs determined according to characteristics including the sex, stage of life, level of physical activity, and anthropometric measures of healthy individuals.

Nutritional status was defined and assessed through anthropometric measurements, including weight (assessed using calibrated digital scales with participants barefoot and in lightweight clothing) and height. Height was measured using a metric tape affixed to a flat wall surface at a 90° angle to the floor, with the participant standing barefoot, heels together, and head in the Frankfort horizontal plane. The principal researcher took these measurements. In cases where direct measurement was not feasible, height was self-reported. If a self-reported value was deemed implausible during the interview (e.g., grossly inconsistent with observed body proportions), it was objectively estimated by the principal researcher using her own height as a visual reference for comparison. The Body Mass Index (BMI) was calculated (weight/height (Pedraza and de Menezes, 2015)) and classified according to WHO (Padovani et al., 2006) criteria.

The current study was conducted in accordance with the ethical guidelines for the protection of human subjects established by Brazil's National Health Council (Resolution No. 466/2012). It was approved by the Institutional Review Board of Santo Amaro University under protocol no. 3.208.096.

### 2.4 Statistical analyses

To characterize energy consumption and macronutrient intake, food intake data collected via 24-h recall were analyzed using the Shapiro-Wilk test to verify the normal distribution of the data. The results were expressed as median, mean, and standard deviation. The percentage of Total Energy Value (% VET) for total proteins, carbohydrates, and lipids was calculated as follows: (energy from the nutrient / total caloric intake) * 100. The adequacy of these percentages, as well as the absolute intake of cholesterol and fiber, was then evaluated by comparison with the recommendations of the Dietary Reference Intakes (DRIs). Vitamin and mineral adequacy were assessed by comparing median and mean intake values to sex- and age-specific DRIs.

The Kruskal-Wallis test was applied to evaluate differences in dietary intake across nutritional status categories. The results of nutritional status comparisons are described narratively in the results section. All statistical tests employed a significance level of α = 0.05 and, the statistical analyses were performed using IBM SPSS V.28 software.

## 3 Results

Among the 160 participants (120 families) across 12 riverside communities, 60.6 % (*n* = 97) were female and 39.4 % (*n* = 63) were male, with a mean age of 40.9 years (range, 18–75). Nutritional status analysis revealed a 59.6 % prevalence of overweight/obesity (overweight: 39.7 % males vs. 36.8 % females; obesity: 17.5 % males vs. 23.2 % females). Two pregnant women were excluded from the BMI classification (Table 1).

**Table 1:** Sociodemographic characteristics, nutritional status, and food consumption profile of riverside communities in the Brazilian Amazon, 2019.

Characteristic	Category	Value
Sociodemographic Data		
Communities	n	12
Families	n	120
Participants	n	160
Female	n (%)	97 (60.6)
Male	n (%)	63 (39.4)
Mean Age	years (range)	40.9 (18–75)
Nutritional Status*		
Overweight/Obesity (Total)	% (n)	59.6 (94)
- Overweight (Men)	% (n)	39.7 (25)
- Overweight (Women)	% (n)	36.8 (35)
- Obesity (Men)	% (n)	17.5 (11)
- Obesity (Women)	% (n)	23.2 (22)
Cooking Fuel Used		
LPG only	% (n)	80.0 (96)
Charcoal only	% (n)	4.2 (5)
Combination of fuels	% (n)	14.2 (17)
Food Group Consumption		
	Consumed % (n)	Adequate Intake % (n)
Oils	100.0 (120)	0 (120)
Meat	100.0 (120)	95.8 (115)
Vegetables	98.4 (118)	84.7 (100)
Legumes	94.2 (113)	13.3 (15)
Fruits	99.2 (119)	62.2 (74)
Dairy Products	97.5 (117)	59 (69)
Cereals/Pasta	21.6 (26)	96.2 (25)
Sweets	89.2 (107)	77.6 (83)
Sausages	58.4 (70)	54.3 (38)

Note: LPG, Liquefied Petroleum Gas. *Two pregnant women were excluded from the nutritional status (BMI) classification.

Of the families studied, 80 % use only liquified petroleum gas (LPG) for food preparation, 4.2 % use only charcoal, and 14.2 % use a combination of fuels. Other findings included universal consumption of oils (100 %) and meat (100 %), high intake of vegetables (98.4 %), fruits (99.2 %), and dairy products (97.5 %), and low consumption of cereals/pasta (21.6 %), with frequent intake of sweets (89.2 %) and sausages (58.4 %). The analysis of dietary adequacy revealed that most families met recommended intake levels for cereals/pasta (96.2 %), meat (95.8 %), and sweets (77.6 %) (Table 1).

Analysis of 24-h dietary recalls (Table 2) revealed that macronutrient distribution aligned with the WHO guidelines (proteins: 17.4 %, carbohydrates: 53.2 %, lipids: 29.6 %), although fiber intake was critically low (10.9 g/day). The micronutrient assessment (Table 3) revealed deficits in vitamins A, D, C, E, and folate, particularly among adults over 50, as well as insufficient levels of calcium, potassium, iron, and zinc (Table 4). Conversely, selenium, magnesium, and sodium intake exceeded recommended levels. To complement the individual intake data, household monthly acquisition of key commodities was assessed. Based on reported purchases, the average monthly per capita consumption was estimated at 1.7 l of oil and 2.78 kg of sugar. These values exceed nutritional recommendations for the Brazilian population (480 ml and 840 g per person per month, respectively) by 30–33 %.

**Table 2:** Daily caloric intake and macronutrient distribution among riverside communities in the Brazilian Amazon, 2019.

Macronutrients/Recommendations	Median	Mean ± SD	% TEV
Calories (Kcal/day)	1588	1686 ± 741	–
Protein (g) 15 to 20 %	69.2	71.5 ± 26.5	17.4
Carbohydrates (g) 50–60 %	211.1	221.6 ± 117.3	53.2
Lipid (g) 25 to 30 %	52.2	55.8 ± 31.4	29.6
Cholesterol (mg) >300 mg/day	140.3	206.2 ± 174.8	–
Saturated fat (mg) >10 %	7.3	7.8 ± 4.9	4.4
Polyunsaturated fat (mg) 6–11 %	11.7	12.9 ± 10.8	7.3
Monounsaturated fat (mg) 10–20 %	9.4	11.4 ± 10.1	6.4
Fiber (g) 25-30 g	8.5	10.9 ± 9.6	–

Note: SD, Standard Deviation; TEV, Total Energy Value. Data presented as median and mean ± standard deviation. Macronutrient distribution adequacy was assessed by comparing it with the Dietary Reference Intakes. The percentage of Total Energy Value was calculated as (energy from nutrient/total energy intake) × 100.

**Table 3:** Daily vitamin intake compared to Dietary Reference Intakes among riverside communities in the Brazilian Amazon, stratified by sex and age group, 2019.

Vitamin	Sex and Age Group	Female				Male			
		18–30 years	31–50 years	51–70 years	>70 years	18–30 years	31–50 years	51–70 years	>70 years
A (mcg/day) RDI = 700/900 μg*	Median	210.8	277.4	248.1	105.8	152.1	205.1	148.5	157.5
	Mean ± SD	492.6 ± 695.2	891.7 ± 1386.4	1152.1 ± 1347.7	115.8 ± 96.3	151.6 ± 52.6	870.0 ± 1119.6	472.6 ± 876.3	165.0 ± 87.8
D (mcg/day) RDI = 5/10/15 μg**	Median	1.85	2.10	1.65	0.80	2.30	2.20	1.20	1.35
	Mean ± SD	1.88 ± 0.9	1.96 ± 1.2	1.37 ± 0.95	1.00 ± 0.58	2.50 ± 0.9	2.00 ± 1.4	1.80 ± 2.4	1.30 ± 0.6
B12 (mcg/day) RDI = 2.4 μg	Median	4.35	3.94	3.60	2.27	4.60	4.20	4.20	4.60
	Mean ± SD	4.17 ± 1.71	3.91 ± 1.9	3.15 ± 2.4	3.62 ± 3.88	4.90 ± 2.3	4.50 ± 3.55	3.80 ± 2.6	4.50 ± 1.7
C (mg/day) RDI = 75/90 mg***	Median	8.50	7.65	14.50	14.80	5.25	10.00	3.50	2.65
	Mean ± SD	73.5 ± 185.1	13.3 ± 18.6	37.2 ± 58.9	14.8 ± 13.2	6.50 ± 4.2	43.3 ± 93.1	3.70 ± 2.9	2.70 ± 2.2
E (mg/day) RDI = 15 mg	Median	21.55	21.20	9.20	4.10	19.20	20.00	17.30	9.85
	Mean ± SD	22.2 ± 15.5	22.7 ± 16.8	16.6 ± 18.0	35.1 ± 53.7	20.6 ± 8.6	22.4 ± 16.7	15.2 ± 12.7	9.10 ± 6.2
K (mcg/day) RDI = 90/120 μg****	Median	1483.7	1277.0	1495.7	2465.8	1276.5	1687.3	1243.3	1464.4
	Mean ± SD	1439.7 ± 445.7	1355.8 ± 532.8	1639.1 ± 722.5	2052.5 ± 963.1	1536.2 ± 748.2	2188.6 ± 2295.8	1224.2 ± 639.1	1511.3 ± 395.7
Folate (mcg/day) RDI = 400 μg	Median	29.0	40.4	28.9	36.8	19.7	35.1	14.0	24.7
	Mean ± SD	36.8 ± 24.9	43.7 ± 33.1	40.8 ± 36.2	42.1 ± 36.7	55.0 ± 80.6	41.8 ± 34.1	32.7 ± 32.3	24.9 ± 19.7

**Note.** Data presented as median and mean ± standard deviation. Dietary Reference Intakes values vary by sex and age: *700 micrograms for women, 900 micrograms for men; **5 micrograms (18–50 years), 10 micrograms (51–70 years), 15 micrograms (>70 years); ***75 mg for women, 90 mg for men; ***90 micrograms for women, 120 micrograms for men.

**Table 4:** Daily mineral intake compared to Dietary Reference Intakes among riverside communities in the Brazilian Amazon, stratified by sex and age group, 2019.

Mineral	Sex and Age Group	Female				Male			
		18–30 years	31–50 years	51–70 years	>70 years	18–30 years	31–50 years	51–70 years	>70 years
Calcium (mg/day) RDI = 800/1000 mg*	Median	319.4	333.4	314.6	708.3	165.5	193.4	232.6	373.2
	Mean ± SD	390.3 ± 270.5	362.8 ± 251.1	337.8 ± 201.0	834.4 ± 363.4	259.7 ± 265.6	245.1 ± 132.4	242.0 ± 163.7	417.6 ± 202.0
Potassium (mg/day) RDI = 2600/3400 mg**	Median	935.6	914.1	887.0	1298.6	930.0	904.6	751.8	946.3
	Mean ± SD	954.6 ± 260.5	899.3 ± 297.6	163.1 ± 62.2	1356.2 ± 588.3	993.9 ± 453.1	959.4 ± 293.8	728.7 ± 257.8	897.6 ± 213.9
Magnesium (mg/day) RDI = 310–420 mg***	Median	115.2	127.8	153.2	171.8	130.2	128.5	113.9	111.2
	Mean ± SD	127.8 ± 40.8	131.1 ± 55.0	9.7 ± 5.6	193.0 ± 117.9	143.0 ± 61.4	140.1 ± 44.4	124.8 ± 75.3	120.9 ± 26.7
Iron (mg/day) RDI = 8–18 mg****	Median	6.8	10.5	8.7	15.7	6.8	11.7	7.3	6.6
	Mean ± SD	7.6 ± 3.0	11.9 ± 7.7	9.7 ± 5.6	13.3 ± 10.0	7.9 ± 5.3	11.9 ± 4.4	9.5 ± 6.3	7.3 ± 4.1
Zinc (mg/day) RDI = 8/11 mg*****	Median	4.5	4.9	5.4	7.5	3.5	5.3	3.7	5.7
	Mean ± SD	5.8 ± 4.0	5.9 ± 4.0	6.1 ± 3.4	8.7 ± 6.9	6.7 ± 6.7	6.9 ± 4.4	5.2 ± 3.7	5.9 ± 4.2
Selenium (mcg/day) RDI = 55 μg	Median	92.5	97.3	96.5	51.3	123.1	115.6	70.5	80.6
	Mean ± SD	94.5 ± 45.7	99.4 ± 49.6	89.1 ± 48.8	45.4 ± 40.1	107.4 ± 60.0	115.5 ± 58.1	84.1 ± 40.5	78.7 ± 23.8
Sodium (mg/day) RDI = 1500 mg	Median	756.6	725.4	856.5	594.8	425.6	1136.6	768.3	371.6
	Mean ± SD	823.0 ± 459.8	946.0 ± 792.4	1197.9 ± 1309.3	489.3 ± 253.2	731.9 ± 841.0	932.3 ± 499.1	757.9 ± 315.9	555.5 ± 418.6

**Note.** Data presented as median and mean ± standard deviation. Dietary Reference Intakes values vary by sex and age: *1000 mg for >50 years, otherwise 800 mg; **3400 mg for men, 2600 mg for women; ***Women: 310 mg (18–30 years), 320 mg (31+ years); Men: 400 mg (18–30 years), 420 mg (31+ years); ****Women: 18 mg (31–50 years), 8 mg otherwise; Men: 8 mg (51+ years), 15 mg (18–50 years); ****8 mg for women, 11 mg for men.

Stratification by nutritional status revealed no statistically significant differences in caloric intake (*p* = 0.17), protein intake (*p* = 0.35), carbohydrate intake (*p* = 0.26), or lipid intake (*p* = 0.33) among BMI categories. Similarly, fiber consumption, cholesterol levels, and fat subtypes (saturated, monounsaturated, and polyunsaturated) showed no meaningful variations across groups.

## 4 Discussion

Riverside populations in Roraima and Amazonas exhibit a nutritional transition marked by high prevalences of overweight (39.7 % in men; 36.8 % in women) and obesity (17.5 % in men; 23.2 % in women), correlating with the reduced use of traditional food systems and increased reliance on industrialized/ultra-processed foods (Mercado et al., 2015; Clímaco et al., 2021; Bezerra et al., 2018). These shifts, along with low intake of fruits and vegetables compared to recommended consumption frequency, raise concerns due to their associations with chronic diseases (Barroso et al., 2017).

Although the majority of families reported consuming vegetables (94.2 %), legumes (98.4 %), fruits (99.2 %), and milk (97.5 %), the consumption frequency in most cases was below five days/week, indicating inadequacy (Table 1). This likely reflects limited market access to perishable foods, consistent with findings from Pantanal communities, where seasonality and climatic conditions affected availability (Clímaco et al., 2021).

Cereals (96.2 % adequacy) and meats (95.8 %) were consumed in adequate amounts, reflecting traditional dietary patterns, as identified by Gama et al. (2022) (Gama et al., 2022; Medeiros and Mainbourg, 2023). However, 58.4 % of households consumed sausages, and 89.2 % consumed sweets/soft drinks—ultra-processed foods linked to obesity and chronic diseases (da Louzada et al., 2015). The average monthly consumption of oil (1.7 L/person) and sugar (2.78 kg/person) exceeded recommendations by 30–33 %, indicating significant dietary shifts. These results show that sugar and oil play an essential role in the local diet, primarily used in fried foods and as a sweetener for juices, açaí, and coffee, indicating trends in food consumption related to the nutritional transition. The increasing prevalence of diet-related diseases may be a reflection of inadequate eating patterns (Nestel and Mori, 2022). Similarly, the same trend was observed in a study by Murrieta et al. (2023) (Murrieta et al., 2023), carried out in two riverside populations of the Amazon.

Macronutrient distribution was inadequate, except for cholesterol, which remained within recommended levels. Crucially, fiber intake (10.9 g/day) fell well below the WHO recommendations (25 g/day), which can be explained by low fruit and vegetable consumption (Table 2). According to the Brazilian Society of Cardiology, recommendations for daily fat intake specify that monounsaturated fats should comprise 20 % and polyunsaturated fats 10 % of total energy intake. At the same time, dietary cholesterol should be limited to <300 mg/day to help control blood cholesterol levels (Izar et al., 2021).

Cardiovascular Disease is the leading cause of death globally, accounting for approximately 30 % of deaths worldwide—a rate similar to that observed in Brazil (Polanczyk, 2020). The primary pathophysiological basis for cardiovascular events is atherosclerosis, a process that develops insidiously over decades, and whose first clinical signs can be fatal or severely debilitating. The formation of atheromatous plaque in blood vessel walls, along with its clinical consequences (such as myocardial infarction and stroke), is closely associated with cardiovascular risk factors, including hypercholesterolemia, hypertriglyceridemia, low HDL—C, systemic arterial hypertension, diabetes mellitus, and obesity. Within this context, diet plays a crucial role in modulating lipid levels, directly influencing the presence and severity of these risk factors (Korakas et al., 2018).

Dietary fiber is primarily obtained from *in-nature* or minimally processed foods, which play a protective role against various chronic diseases and gastrointestinal disorders. Given its benefits, the World Health Organization (WHO) recommends a minimum dietary fiber intake of 25 g per 2000 kcal (or 12.5 g per 1000 kcal) (Martins et al., 2023), a guideline also adopted by the Brazilian Ministry of Health (Brasil. Ministério da Saúde. Secretaria de Atenção à Saúde, 2014).

In the current study, the average fiber intake (10.9 g) among the riverside population was below the WHO reference value, which can be attributed to the low consumption of fruits and vegetables compared to recommended consumption frequency. These results are consistent with those of Cruz et al. (2021) (Cruz et al., 2021), who reported that most Brazilians have insufficient intake of fiber and confirmed the unfavorable nutritional profile of ultra-processed foods in terms of fiber content, highlighting their negative impact on overall dietary quality.

Significant vitamin deficiencies (A, D, C, folate, K) were observed, related to the low vegetable intake (Table 3). Although fish is an excellent source of vitamins D and B12, the amounts ingested were inadequate to meet the recommended intake. The adequacy of vitamin E intake in comparison to the deficiencies observed in other vitamins can be explained by the high consumption of Brazil nuts by this population.

Most minerals (Table 4) - including calcium, potassium, magnesium, iron, and zinc - showed inadequate intake, which was again linked to low fruit/vegetable consumption compared to recommended consumption frequency. Vitamin D deficiency likely further impaired calcium absorption. Insufficient quantities of these foods in the diet can contribute to intake below the recommended level (Maurya and Aggarwal, 2017).

Selenium was the only mineral that met the recommendations of the DRIs, which can be explained by the fact that daily needs are generally lower compared to other minerals, making it easier to reach the appropriate levels, and also not depending so much on other nutrients for absorption and use by the body. Another explanation for the adequacy of selenium can be directly attributed to the consumption of Brazil nuts, which are one of the richest sources of selenium. A relatively small amount of this food can provide a significant amount of the mineral, often exceeding the recommended daily needs. In a survey conducted by Martens et al. (2015) (Martens et al., 2015), schoolchildren in the Amazon region presented adequate intake of this mineral, and supplementation with Brazil nuts, led to excess levels of selenium.

Notably, caloric and macronutrient intake showed no significant differences across nutritional status groups (*p* > 0.05), suggesting that food quality and meal distribution may influence weight more than total intake. The high prevalence of obesity reflects increasing industrialized food consumption and nutritional transition patterns similar to those in other developing regions.

When investigating the reasons for the high prevalence of overweight and obesity (59.6 %) in this study, it was found that the population's diet included a significant proportion of industrialized foods, recognized as a risk factor (including soft drinks, snacks, fried foods, and sugars). There is a growing trend of overweight and obesity in the communities studied, showing that they are undergoing a nutritional transition process that is characteristic of third-world countries (Murrieta et al., 2023). Our findings align with those of Silveira et al. (2023) (Silveira et al., 2023b), who also reported nutritional imbalances in riverside populations. However, while that study primarily documented anthropometric outcomes, our analysis further elucidates the underlying dietary constraints—namely, low access to and consumption of a diverse range of healthy foods—that explain these imbalances.

The decline in traditional Amazonian foods in family meals can be attributed in part to changes in livelihoods and lifestyles, as well as deforestation, which has impacted the availability of wild game, fruits, and nuts (Oestreicher et al., 2020).

Study limitations include the inability of the 24-h recall to capture long-term patterns, although the additional use of the FFQ mitigated this concern. in addition, the cross-sectional design precludes causal inference, and data collection during the flood season likely affected food availability. However, the study was able to elucidate some complex relationships in food consumption, going beyond the mere estimation of total calories or isolated nutrient intake, which makes this limitation less significant. Future research should consider seasonal variations in riverside diets.

## 5 Conclusion

Although caloric and macronutrient intake did not differ significantly between nutritional status groups, the general diet was low in variety, fiber, vitamins, and micronutrients. Seasonal variations and traditional practices must be considered in order to better understand the determinants of nutritional health. The findings underscore the importance of interventions that promote dietary diversity, enhance nutrition, improve food security, and preserve local practices in riverside communities. Public policies that ensure access to fresh, nutritious food are crucial for addressing these challenges.

## CRediT authorship contribution statement

**Patrícia Colombo-Souza:** Writing – review & editing, Writing – original draft, Validation, Formal analysis, Data curation, Conceptualization. **Julia de Macedo Moura Silva:** Writing – review & editing, Writing – original draft, Data curation, Conceptualization. **Bruna da Silva Miranda:** Writing – review & editing, Writing – original draft, Data curation. **Ana Paula Ribeiro:** Writing – review & editing, Methodology, Data curation. **Marco Antonio Zonta:** Writing – review & editing, Writing – original draft, Validation.

## Author statement

We declare that this manuscript is original, has not been published before, and is not currently being considered for publication elsewhere. We confirm that the manuscript has been read and approved by all named authors, and there are no other persons who satisfy the criteria for authorship. We further confirm that all authors have approved the order of authors listed in our manuscript. We understand that the Corresponding Author is the primary contact for the editorial process. She is responsible for communicating with the other authors regarding progress, revisions, and the final approval of proofs.

## Funding

There was no funding.

## Declaration of competing interest

The authors declare that they have no known competing financial interests or personal relationships that could have appeared to influence the work reported in this paper.

## Data Availability

Data will be made available on request.
